# A Sane Christmas

**Published:** 1911-12

**Authors:** 


### A Sane Christmas

WE wish our readers a Merry Christmas. Let us remember that there is something more to the Christmas spirit than mere giving and receiving, merriment, and overeating and overdrinking. Some men show their Christmas spirit by pinning mistletoe to their shoes. The country has with surprising rapidity, developed a relatively sane Fourth of July. It is equally important to develop a sane Christmas. The former has been marked by a decided diminution of casualties, especially of tetanus. Let us not forget that, next after the diminution of infectious disease and direct risk of violence, conservation of health and life depends upon diminishing the general wear and tear of existence. It is not improbable that Christmas, as at present celebrated, causes more deaths from exposure and fatigue among postal and express employees, clerks and others, than have ever been due to fire works.

Aside from this, it is worth considering that a hysteric extravagance in purchases, largely of useless articles, concentrated within a few weeks of the year, disturbs normal business, produces poverty and suffering, and ultimately costs health and life. Many of us have witnessed the passing away of the old-time, kindly visiting on New Year's day, because the custom reached a point of intolerable excess. It would be a pity to lose the Christmas observation too. Let us be warned in time and remember that Christmas is a day sacred to the home, the children, and the poor.

Many a shrewd physician has discontinued the natural issuance of bills at the beginning of the year, realizing that Santa Claus has levied so high a tax on his clientele that he will scarcely collect enough within the month to pay for the postage. This is an accurate index of the economic disturbance brought about by the tendency to excess in Christmas giving. It is not stinginess but true generosity to demand a sane spirit at Christmas, to eliminate extravagance, and sacrifice of family duty and charity to the worthy poor, on the altar of social ostentation.

